# Classification of Sharks in the Egyptian Mediterranean Waters Using Morphological and DNA Barcoding Approaches

**DOI:** 10.1371/journal.pone.0027001

**Published:** 2011-11-02

**Authors:** Marie Moftah, Sayeda H. Abdel Aziz, Sara Elramah, Alexandre Favereaux

**Affiliations:** 1 Zoology Department, Faculty of Science, Alexandria University, Alexandria, Egypt; 2 Oceanography Department, Faculty of Science, Alexandria University, Alexandria, Egypt; 3 University de Bordeaux, IINS, UMR 5297, F-33000 Bordeaux, France; 4 CNRS, IINS, UMR 5297, F-33000 Bordeaux, France; Biodiversity Insitute of Ontario - University of Guelph, Canada

## Abstract

The identification of species constitutes the first basic step in phylogenetic studies, biodiversity monitoring and conservation. DNA barcoding, i.e. the sequencing of a short standardized region of DNA, has been proposed as a new tool for animal species identification. The present study provides an update on the composition of shark in the Egyptian Mediterranean waters off Alexandria, since the latest study to date was performed 30 years ago, DNA barcoding was used in addition to classical taxonomical methodologies. Thus, 51 specimen were DNA barcoded for a 667 bp region of the mitochondrial *COI* gene. Although DNA barcoding aims at developing species identification systems, some phylogenetic signals were apparent in the data. In the neighbor-joining tree, 8 major clusters were apparent, each of them containing individuals belonging to the same species, and most with 100% bootstrap value. This study is the first to our knowledge to use DNA barcoding of the mitochondrial *COI* gene in order to confirm the presence of species *Squalus acanthias*, *Oxynotus centrina*, *Squatina squatina*, *Scyliorhinus canicula*, *Scyliorhinus stellaris*, *Mustelus mustelus*, *Mustelus punctulatus* and *Carcharhinus altimus* in the Egyptian Mediterranean waters. Finally, our study is the starting point of a new barcoding database concerning shark composition in the Egyptian Mediterranean waters (Barcoding of Egyptian Mediterranean Sharks [BEMS], http://www.boldsystems.org/views/projectlist.php?&#Barcoding%20Fish%20%28FishBOL%29).

## Introduction

There are probably close to 30 000 fish species worldwide, constituting about 50% of all vertebrate species (www.fishbase.org). They are systematically very diverse, ranging from ancient jawless species (Agnatha) to cartilaginous fishes (Chondrichthyes) and bony fish (Osteichthyes) [1]. Cartilaginous fishes (sharks, rays, skates, and chimaeras) are the phylogenetically oldest group of living jawed vertebrates. They are an important out-group for understanding the evolution of bony vertebrates such as teleost fishes and human [2].

The identification of species constitutes the first basic step for biodiversity monitoring and conservation [3]. Fish species identification mainly relies on morphometric and meristic characteristics [4]. However, there are pitfalls in relying primarily on morphology when attempting to identify fishes during various stages of their development not considered in original treatments or when examining fragmentary, partial or processed remains. Even when intact adult specimens are available, the morphological characteristics used to discern species can be so subtle that identification is difficult even for trained taxonomists [5].

It has been recently proposed that the use of DNA methods can circumvent such a problem [6]. The reconstruction of phylogenetic relationships based on molecular data in addition to the classical methodologies has helped to resolve taxonomic uncertainties for fishes [7]–[9]. The rise in molecular biological techniques in marine forensic science has facilitated the development of accurate taxonomic identification of shark species by sampling biological tissue [10]–[14].

DNA barcoding, i.e. the sequencing of a short standardized region of DNA, has been proposed as a new tool for animal species identification [15]. The technique uses universal primers to amplify an approximately 650 bp-long region of the mitochondrial *cytochrome c oxidase I* (*COI*) gene. This region is sequenced to provide the DNA barcode for the specimen under study, and is compared to barcodes from reference specimens to obtain a species identification. Within-species variation for this gene is low compared with between-species variation. As a consequence, species are regularly delineated by a particular sequence or by a tight cluster of very similar sequences [5]. DNA barcoding has enabled discrimination of 98–99% of fish species examined to date, and its power to discriminate closely related species is largely attributable to the abundance of synonymous nucleotide changes [16].

Several studies have been done on the composition of shark species in different regions of the Mediterranean Sea [17]–[19]. However, shark species composition in the Egyptian Mediterranean waters is still scarcely known. To our knowledge, the first study was done by Mazhar (1974), who studied the taxonomy and anatomy of the sharks and rays in the area [20]. Then, the Institute of Oceanography and Fisheries in Alexandria revised the shark taxonomy of some shark families [Marine biological reference collection project (1978–1979)]. The most recent study in the area was by Hosny (1981), who studied the biological aspects of the Triakidae family and shark species composition off Alexandria [21].

Our investigation intended to provide an update on shark composition in the area since the latest study to date was performed 30 years ago. In addition to classical taxonomical methodologies, we used barcoding tools and initiated a new barcoding database concerning shark composition in the Egyptian Mediterranean waters.

## Materials and Methods

### Ethics statement

All experiments were carried out on dead shark specimens caught by local fishermen during fishing campaigns. Therefore, the local ethics committee deemed that approval was not necessary.

### Specimens

Fifty-one (51) shark specimens belonging to 6 families were collected from the commercial catch received in the two major fish markets in Alexandria, namely Abu Qir Fishing Centre and Ras-el-Tin Fishing Centre (Anfoushi). The fishing boats were operating in Alexandrian waters from longitude 29°40′E to 30°20′E, and the period of sample collection was from May to November 2008.

### Classical approach

In order to identify and classify sharks using the traditional standard methods, we followed the recommendations proposed in previous studies [22]–[24]. Morphometric measurements were performed for each shark specimen and expressed as absolute values and ratio indices of total length (TL) or head length (HDL). Meristic measurements included gill slit count, dorsal fin number and spines (if found) and total number of vertebrae. However, only the following measurements are presented in this paper; Fork length (FL), Precaudal-fin length (PCL), Predorsal-fin length (PD), Prepectoral-fin length (PP1), Prepelvic-fin length (PP2), Preanal-fin length (PAL), Body depth (BD), Head length (HDL), Preorbital length (POB) and Eye diameter (ED).

### Molecular approach

Muscle tissue samples were dissected from shark specimens and were kept frozen at −80°C until DNA extraction experiments. Approximately 2 cm^3^ of muscle tissue sample was sub-sampled for DNA extraction. For PCR amplification of mitochondrial *COI*, we used previously described [1] primers (Fish F1, Fish R1, Fish F2, Fish R2). Then, DNA was re-extracted from the gel using QIAEX (Qiagen, Germany), or PCR reaction products were purified using Agencourt® AM Pure® Protocol. The resulting DNA was then checked for its amount and purity using a Nanodrop spectrophotometer (Nanodrop ND-1000, Thermo Fisher Scientific, USA). Products were labeled using the Big Dye® Terminator v.1.1 Sequencing Kit (Perkin Elmer Applied Biosystems, USA), and sequenced using an automated sequencer ABI 33130x1 (Applied Biosystems, HITACHI, Japan). Sequencing was performed in triplicate for each specimen (Test no. 1, 2 and 3).

Various software was used to analyze sequences: Finch TV Version 1.4.0, Geospiza In.; BioEdit Sequence alignment Editor, Tom Hall, and MEGA5 [25]. All new data were deposited in GenBank (http://www.ncbi.nlm.nih.gov/genbank/) and BOLD (http://www.barcoding) databases.

For subsequent procedures, a consensus sequence was determined for each specimen. Sequence divergence values within species, within genera, within orders and within classes were calculated using MEGA5 free software [25], where the Kimura 2 Parameters (K2P) model was chosen as distance model [26].

A neighbor-joining (NJ) tree of K2P distance was constructed to provide a graphical representation of the patterning of divergence between the specimens [27]. The NJ tree was then confirmed by bootstrapping to assign confidence levels to each branch in the tree.

## Results

Fifty-one (51) shark specimens were collected and studied using classical and molecular approaches. Difficulties in collecting samples were reflected in the low number of specimens under study. This is mainly due to the irregular supply of sharks in the fishing centres because of their low commercial value.

### *I-* Morphological and biometric analyses

In order to identify the specimens, keys and diagnostic features to orders, families, genera and species determined by the *Food and Agriculture Organization* were followed. This method was combined with molecular approaches in order to determine the species identification for each specimen. The main morphological measurements are summarized in Table 1 together with the species identifications.

**Table 1:** Morphological measurements.

Species	Sample no.	FL/TL%	PCL/TL%	PD/TL%	PP1/TL%	PP2/TL%	PAL/TL%	BD/TL%	HDL/TL%	POB/HDL%	ED/HDL%
*M. mustelus*	1–4, 9, 12–14	85.47±0.37	78.88±0.43	27.66±0.99	18.08±0.94	42.68±1.21	63.93±0.82	7.53±1	19.68±0.88	34.71±1.45	0.99±0.13
*M. punctulatus*	5–8, 10–11	85.88±0.21	79.31±0.26	28.11±0.34	17.72±0.61	42.87±0.72	63.5±0.87	6.81±0.48	18.82±0.49	37.12±1.14	1.07±0.07
*S. acanthias*	15–19	87.91±0.9	79.99±1.07	28.52±0.73	19.83±0.9	45.53±0.95	-	8.99±1.06	20.64±0.34	29.71±0.98	1.54±0.13
*S. stellaris*	20–23	-	78.55±0.49	49.57±0.73	16.79±0.33	42.18±0.5	59.37±0.61	8.14±0.89	18.42±0.67	26.3±1.48	0.72±0.04
*S. canicula*	24–45	-	78.69±0.97	49.04±0.88	15.54±0.66	38.39±0.96	57.73±1.22	6.82±0.57	16.79±0.65	25.01±2.57	0.69±0.12
*S. squatina*	46–48	95.17±0.45	84.32±0.54	62.37±0.53	18.57±0.4	38.13	-	7.22±0.26	19.12±0.62	-	4.73±0.45
*C. altimus*	49–50	80.43	72.87	27.96	19.58	49.75	6.45	13.13	21.75	33.98	8.43
*O. centrina*	51	-	78.49	22.38	17.21	59.55	-	16.35	17.56	23.53	15.2

Classical taxonomic measurements represented by the mean and standard deviation, except for species represented by fewer than 3 specimens, where only the mean is calculated. Total length (TL), Head length (HDL), Fork length (FL), Precaudal-fin length (PCL), Predorsal-fin length (PD), Prepectoral-fin length (PP1), Prepelvic-fin length (PP2), Preanal-fin length (PAL), Body depth (BD), Head length (HDL), Preorbital length (POB), Eye diameter (ED).

### II- GenBank and BOLD matching

The mitochondrial *COI* gene was amplified and sequenced in triplicate for each specimen (n = 51), with an average length of 667 bp; BOLD identification numbers and GenBank accession numbers are summarized in Table 2. Sequences were matched for their maximum identity with those available in the GenBank database (http://www.ncbi.nlm.nih.gov/genbank/), and gave matches to shark species for 93% with an average maximum identity of 98%. In addition, sequences were analyzed the using BOLD identification engine (Barcode Of Life Data system, version 2.5 http://www.barcodinglife.org). This gave matches to shark species for 84.83%, with an average similarity of 99% (Table S1).

**Table 2:** BOLD identification numbers and GenBank accession numbers.

Species	BOLD ID	GenBank Accession Number
*Carcharhinus altimus*	FMS050-10	JN641206
*Carcharhinus altimus*	FMS049-10	JN641207
*Mustelus mustelus*	FMS014-10	JN641208
*Mustelus mustelus*	FMS013-10	JN641209
*Mustelus mustelus*	FMS012-10	JN641210
*Mustelus mustelus*	FMS009-10	JN641211
*Mustelus mustelus*	FMS004-10	JN641212
*Mustelus mustelus*	FMS003-10	JN641213
*Mustelus mustelus*	FMS002-10	JN641214
*Mustelus mustelus*	FMS001-10	JN641215
*Mustelus punctulatus*	FMS011-10	JN641216
*Mustelus punctulatus*	FMS010-10	JN641217
*Mustelus punctulatus*	FMS008-10	JN641218
*Mustelus punctulatus*	FMS007-10	JN641219
*Mustelus punctulatus*	FMS006-10	JN641220
*Mustelus punctulatus*	FMS005-10	JN641221
*Oxynotus centrina*	FMS051-10	JF834320
*Scyliorhinus canicula*	FMS045-10	JN641222
*Scyliorhinus canicula*	FMS044-10	JN641223
*Scyliorhinus canicula*	FMS043-10	JN641224
*Scyliorhinus canicula*	FMS042-10	JN641225
*Scyliorhinus canicula*	FMS041-10	JN641226
*Scyliorhinus canicula*	FMS040-10	JN641227
*Scyliorhinus canicula*	FMS039-10	JN641228
*Scyliorhinus canicula*	FMS038-10	JN641229
*Scyliorhinus canicula*	FMS037-10	JN641230
*Scyliorhinus canicula*	FMS036-10	JN641231
*Scyliorhinus canicula*	FMS035-10	JN641232
*Scyliorhinus canicula*	FMS034-10	JN641233
*Scyliorhinus canicula*	FMS033-10	JN641234
*Scyliorhinus canicula*	FMS032-10	JN641235
*Scyliorhinus canicula*	FMS031-10	JN641236
*Scyliorhinus canicula*	FMS030-10	JN641237
*Scyliorhinus canicula*	FMS029-10	JN641238
*Scyliorhinus canicula*	FMS028-10	JN641239
*Scyliorhinus canicula*	FMS027-10	JN641240
*Scyliorhinus canicula*	FMS026-10	JN641241
*Scyliorhinus canicula*	FMS025-10	JN641242
*Scyliorhinus canicula*	FMS024-10	JN641243
*Scyliorhinus stellaris*	FMS023-10	JN641244
*Scyliorhinus stellaris*	FMS022-10	JN641245
*Scyliorhinus stellaris*	FMS021-10	JN641246
*Scyliorhinus stellaris*	FMS020-10	JN641247
*Squalus acanthias*	FMS019-10	JN641248
*Squalus acanthias*	FMS018-10	JN641249
*Squalus acanthias*	FMS017-10	JN641250
*Squalus acanthias*	FMS016-10	JN641251
*Squalus acanthias*	FMS015-10	JN641252
*Squatina squatina*	FMS048-10	JN641253
*Squatina squatina*	FMS047-10	JN641254
*Squatina squatina*	FMS046-10	JN641255

Complete list of specimens with corresponding BOLD identification numbers and accession numbers in GenBank database.

Order Squaliformes, family Squalidae, genus *Squalus* was represented by *Squalus acanthias* (Figure S1A). The average of first best similarity for this species using BOLD identification engine was 99.74%. Only one specimen out of 8 could not be clearly identified at the species level with the BOLD engine and the suggested identifications were *Squalus blainville* or *Squalus acanthias*. In such cases, morphological analysis was helpful to confirm specimens as being *Squalus acanthias*. Divergence distance average within species (conspecific distance) was 0.35% (ranging from 0.00 to 0.53%).

Within the same order Squaliformes, family Oxynotidae, genus *Oxynotus* was represented by one species (*Oxynotus centrina*) and only one specimen was collected from the eastern coast of Alexandria (Figure S1B). GenBank matching misidentified this sample, establishing a maximum identity of 92% with *Centroscymnus coelolepis*. However, the BOLD identification engine clearly determined this specimen as being *Oxynotus centrina* with 100% similarity (described as solid identification results).

Order Squatiniformes, family Squatinidae, genus *Squatina* was represented by 3 specimens, belonging to *Squatina squatina* (Figure S1C). Captured specimens were collected from a commercial catch landed at Ras-el-Tin (western Alexandria). These specimens gave a maximum identity of 95.33% when matched with the GenBank database, and 99.38% using the BOLD identification engine. All results of this species were described by the BOLD engine as solid.

Order Carcharhiniformes, family Scyliorhinidae, genus *Scyliorhinus* was represented by two species: *Scyliorhinus canicula* and *Scyliorhinus stellaris* (Figure S1D, E). Specimens of both species were collected from the Ras-el-Tin fishing centre. GenBank matching appropriately identified *S. canicula* and *S. stellaris with* a maximum identity of 97.88% and 98.3% respectively. The BOLD engine gave a solid identification for all samples expected to belong to *S. stellaris*. In contrast, BOLD misidentified 6 out of the 22 specimens belonging to *S. canicula*. Conspecific distance within *S. canicula* showed a divergence distance of 0.34% while those belonging to *S. stellaris* showed a distance of 0.00%. Congeneric distance between *S. canicula* and *S. stellaris* was 7.83% (ranging from 7.67 to 8.07%).

Within the same order Carcharhiniformes, family Triakidae, genus *Mustelus* was represented by *Mustelus mustelus* and *Mustelus punctulatus* (Figure S1F, G). When matched with the GenBank database, specimens belonging to *M. mustelus* species gave 98.22% maximum identity on average. In contrast, specimens belonging to *Mustelus punctulatus* could not be identified at the species level with GenBank (average identity of 98.31% with *Mustelus sp.*). The BOLD engine confirmed the results of GenBank for the samples belonging to *M. mustelus* (average first best matching of 99.71%). Moreover, for the rest of the specimens belonging to genus *Mustelus*, BOLD gave first best similarity matches with *M. punctulatus*, with an average of 99.84%. Using divergence distance, we found that *M. mustelus* and *M. punctulatus* had a conspecific distance of 0.16% and 0.09% respectively. Congeneric distance between *M. mustelus* and *M. punctulatus* was 8.86%.

Finally, within the same order Carcharhiniformes, family Carcharhinidae, genus *Carcharhinus* was represented by 2 specimens that were difficult to identify (Figure S1H). When matched in the Genbank database, both specimens showed a 99% maximum identity with *Carcharhinus altimus*, followed by *Carcharhinus plumbeus* with the same percentage. However, when matched using BOLD identification, both specimens gave *C. plumbeus* as first and second best similarities. In addition the BOLD database stated that species level identification could not be performed. As an alternative identification, BOLD suggested *C. altimus*. These two species are considered as highly related and therefore difficult to distinguish [5]. In order to identify these two specimens at the species level, we used the traditional morphological approaches which identified them both as *C. altimus*.

### III- Neighbor-Joining tree

**Figure 1:**
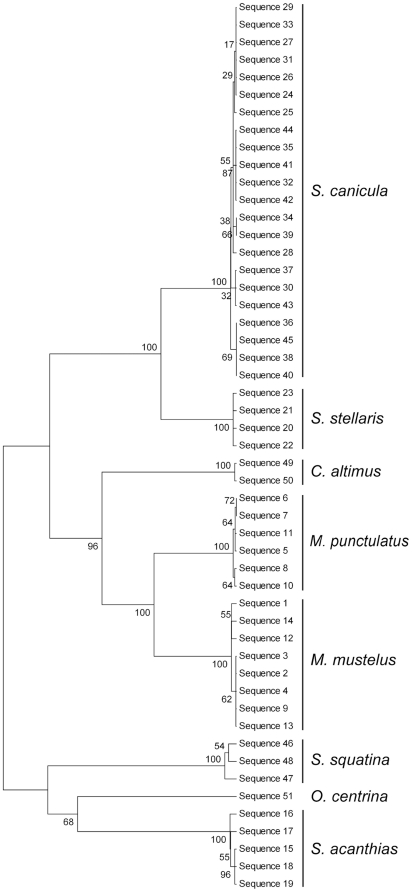
Neighbor-joining tree of 51 COI gene sequences from 8 shark species using K2P distances (bootstrapping values included).

The Neighbor-Joining tree method is conceptually related to clustering, but without assuming a clock-like behavior. Although this study sought only to delaminate species boundaries, there is clearly a phylogenetic signal in the *COI* sequence data. In the resulting NJ tree, the major branches of the tree represent the Superorder Squalimorphi including the orders Squaliformes and Squatiniformes and the Superorder Galeomorphi including the order Carcharhiniformes (Figure 1). Moreover, each of the major clusters in the constructed K2P/NJ tree are composed of individuals from the same species.

It has been suggested that combining NJ and bootstrap analysis [28] is the best way to evaluate trees using distance methods [29]. All clades including individuals belonging to the same species had a 100% bootstrap value. The only exception was the specimen representing the family Oxynotideae, species *Oxynotus centrina* (67%), which was placed on a branch next to the family Squalidae (Figure 1, Table S1).

### IV- Final identification

Using maximum identity with the GenBank database, BOLD identification data, K2P divergence distances, NJ tree bootstrapping and traditional morphological approaches, we were able to assign each specimen and its associated *COI* sequence to a particular species (Figure 1). Thus, our results demonstrate that three orders were recorded in the Mediterranean waters off Alexandria: Squaliformes, Squatiniformes, and Carcharhiniformes. Specimens were found to belong to species: *Squalus acanthias*, *Oxynotus centrina*, *Squatina squatina*, *Scyliorhinus canicula*, *Scyliorhinus stellaris*, *Mustelus mustelus*, *Mustelus punctulatus*, and *Carcharhinus altimus*.

## Discussion

DNA barcoding is a novel system designed to provide rapid, accurate and automatable species identifications using short, standardized gene regions as internal species tags. This was achieved here, where molecular approaches confirmed classical morphological and biometric (morphometric and meristic) methods. The lack of stop codons is consistent with all amplified sequences being functional mitochondrial *COI* sequences. These sequences were about 667 bp in length, suggesting that nuclear DNA sequences originating from mtDNA sequences (NUMTs) were not sequenced (vertebrate NUMTs are typically smaller than 600 bp [30]). Matching sequences with online databases has been considered to date as the simplest way to identify an unknown specimen. In the present study, the GenBank database was useful in giving reliable matching results, which were confirmed by our classical morphological and morphometric approaches. On the other hand, matching using the BOLD identification engine was specific, since it compares each sequence with that of the same gene region in the database.

The Fish Barcode of Life Initiative (Fish-BOL; http://www.fishbol.org) is a concerted global effort to assemble a standardized reference sequence library for all fish species, i.e. one that is derived from voucher specimens with authoritative taxonomic identification. Many of the barcoded fish species uploaded concern marine fish from Australia and Asia, whereas in Europe 440 out of 2028 species had been barcoded until November 2010. Our study is the first to barcode sharks in the Egyptian Mediterranean waters. We used classical taxonomic approaches combined with molecular methods to barcode eight shark species and initiate a new barcoding database called the Barcoding of Egyptian Mediterranean Sharks [BEMS].

The species *Squalus acanthias* has been used as a model for elasmobranch in some DNA sequencing studies [31]–[33]. Ward *et al.* [1], used the DNA barcoding technique to study genetic differentiation among species of the genus *Squalus*, where the *COI* data clearly supported the biological evidence for *Squalus acanthias*.

*Oxynotus centrina* was represented by only one specimen, confirming a previous study mentioning the rarity of this species in the Mediterranean Sea [34].

Recently, the *Squatina squatina COI* gene was used as mitochondrial marker in a comprehensive phylogenetic reconstruction study of 17 *Squatina* species, where the phylogenetic reconstructions were used to test biogeographic patterns. In addition, a molecular clock analysis was conducted to estimate the divergence times of the emerged clades. All analyses showed *Squatina* to be monophyletic [35].

*Scyliorhinus canicula* and *Scyliorhinus stellaris* were clearly distinguished using molecular approaches. The main characteristics that were used to morphologically distinguish between these two related species were the distribution pattern of the colored spots on the skin, and the distinct shape of the anterior nasal flap.

Concerning two other closely related species, *M. punctulatus* and *M. mustelus*, the presence of black spots on the skin is thought to be a distinct characteristic of *M. punctulatus* [22], [23]. However, none of our specimens displayed it, but we noticed that the black bars at the margins of the dorsal fins could be used as a specific characteristic of *M. punctulatus*.

Concerning the genus *Carcharhinus*, we had some difficulty in using matching engines to distinguish between the two closely related species *C. altimus* and *C. plumbeus*. The genus *Carcharhinus* comprises 30 species, 27 of which are barcoded, and some of these such as *C. altimus* and *C. plumbeus*, are known to be very closely related [5]. This was confirmed by Heist and Gold [10], who demonstrated that there is more substitution in the cytochrome-b of the mtDNA observed between Atlantic and Pacific specimens of *C. plumbeus* than between Atlantic specimens of *C. plumbeus* and *C. altimus*. In such instances, *COI* may not enable rigorous species discrimination so an additional marker with a higher rate of evolution might be required.

Although identification trees based on *COI* sequence divergence are not primarily a phylogenetic tool, they do signal some deeper relationships [1]. This was confirmed here since species in a genus and genera in a family generally formed cohesive clusters.

In the present study, all clades including individuals belonging to the same species had a 100% bootstrap value. There was only one exception, specimen number 51, which was represented by 67% bootstrap. Such a low percentage might be due to the fact that the species was represented by only one specimen.

This study is the starting point of a new barcoding database related to shark composition in the Egyptian Mediterranean waters (Barcoding of Egyptian Mediterranean Sharks [BEMS], http://www.boldsystems.org/views/projectlist.php?&#%20Fish%20%28FishBOL%29). It can also be considered as an update of the shark composition list in the area since the latest study to date was performed 30 years ago. Thus, we barcoded and confirmed the presence of the following species: *Squalus acanthias*, *Oxynotus centrina*, *Squatina squatina*, *Scyliorhinus canicula*, *Scyliorhinus stellaris*, *Mustelus mustelus*, *Mustelus punctulatus*, and *Carcharhinus altimus*. Further insights on relationships will be obtained as taxa coverage expands. Future studies over a longer period of time and with more collected specimens are needed to fully describe the shark composition in this area.

## Supporting Information

Figure S1 **Specimens' pictures for each species under study.** A: *Squalus acanthias*; B: *Oxynotus centrina*; C: *Squatina squatina*; D: *Scyliorhinus canicula*; E: *Scyliorhinus stellaris*; F: *Mustelus mustelus*; G: *Mustelus punctulatus* and H: *Carcharhinus altimus*.
(PDF)

Table S1 **Sequence analysis results: maximum identity using GenBank database, first and second best similarities using BOLD identification engine.** X: indicates specimen checked for best match using BOLD identification engine, and the site could not match and diverted it to GenBank database. Consensus identification was decided depending on best similarity for the specimen, using both GenBank database and BOLD identification engine, putting into consideration the morphological and morphometric measurements.
(PDF)

## References

[1] Ward RD, Zemlak TS, Innes BH, Last PR, Hebert PD (2005). DNA barcoding australia's fish species.. Philos Trans R Soc Lond B Biol Sci.

[2] Venkatesh B, Kirkness EF, Loh YH, Halpern AL, Lee AP (2007). Survey sequencing and comparative analysis of the elephant shark (*Callorhinchus milii*) genome.. PLoS Biol.

[3] Dayrat B (2005). Towards integrative taxonomy.. Biol J Linn Soc.

[4] Strauss R, Bond C, Schreck CB, Moyle PB (1990). Taxonomic methods: morphology.. Methods for fish biology.

[5] Ward RD, Hanner R, Hebert PD (2009). The campaign to DNA barcode all fishes, fish-bol.. J Fish Biol.

[6] Hebert PD, Gregory TR (2005). The promise of DNA barcoding for taxonomy.. Syst Biol.

[7] Hanel R, Sturmbauer C (2000). Multiple recurrent evolution of trophic types in northeastern atlantic and mediterranean seabreams (Sparidae, Percoidei).. J Mol Evol.

[8] Craig MT, Pondella DJ, Franck JP, Hafner JC (2001). On the status of the serranid fish genus Epinephelus: evidence for paraphyly based upon 16s rDNA sequence.. Mol Phylogenet Evol.

[9] de la Herran R, Rejon CR, Rejon MR, Garrido-Ramos MA (2001). The molecular phylogeny of the Sparidae (pisces, perciformes) based on two satellite DNA families.. Heredity.

[10] Heist EJ, Gold JR (1999). Genetic identification of sharks in the U.S. atlantic large coastal shark fishery.. Fish Bull.

[11] Lavery S, Pepperell JG (1992). Electrophoretic analysis of phylogenetic relationships among australian Carcharhinid sharks.. Sharks: biology and fisheries.

[12] Martin AP (1991). Application of mitochondrial DNA sequence analysis to the problem of species identification of sharks. in conservation biology of Elasmobranchs.. NOAA Tech Rep NMFS.

[13] Ward RD, Holmes B, White WT, Last PR (2008). DNA barcoding australasian Chondrichthyans: results and possible uses in conservation.. Mar Freshwater Res.

[14] Holmes B, Dirk S, Ward RD (2009). Identification of shark and ray fins using DNA barcoding.. Fish Res.

[15] Hebert PD, Cywinska A, Ball SL, de Waard JR (2003). Biological identifications through DNA barcodes.. Proc Biol Sci.

[16] Ward RD, Holmes BH (2007). An analysis of nucleotide and amino acid variability in the barcode region of cytochrome c oxidase i (cox1) in fishes.. Mol Ecol Notes.

[17] Renon P, Colombo MM, Colombo F, Malandra R, Biondi PA (2001). Computer-assisted evaluation of isoelectric focusing patterns in electrophoretic gels: identification of smoothhounds (*Mustelus mustelus*, *Mustelus asterias*) and comparison with lower value shark species.. Electrophoresis.

[18] Blanco M, Pérez-Martín RI, Sotelo CG (2008). Identification of shark species in seafood products by forensically informative nucleotide sequencing (fins).. J Agric Food Chem.

[19] Iglésias SP, Lecointre G, Sellos DY (2005). Extensive paraphylies within sharks of the order Carcharhiniformes inferred from nuclear and mitochondrial genes.. Mol Phylogenet Evol.

[20] Mazhar FM (1974). The Elasmobranchs of the mediterranean. iv-the spiny dogfish, *Squalus fernandinus*.. Bull Inst Ocean & Fish ARE.

[21] Hosny CF (1981). Studies on fishes of family Triakidae off alexandria..

[22] Compagno LJV (2001). Sharks of the world, an annotated and illustrated catalogue of shark species known to date - bullhead, mackerel & carpet sharks..

[23] Serena F (2005).

[24] Froese R, Pauly D (2009). http://www.fishbase.org.

[25] Tamura K, Dudley J, Nei M, Kumar S (2007). Mega4: molecular evolutionary genetics analysis (mega) software version 4.0.. Mol Biol Evol.

[26] Kimura M (1980). A simple method for estimating evolutionary rates of base substitutions through comparative studies of nucleotide sequences.. J Mol Evol.

[27] Saitou N, Nei M (1987). The neighbor-joining method: a new method for reconstructing phylogenetic trees.. Mol Biol Evol.

[28] Felsenstein J (1985). Confidence limits on phylogenies: an approach using the bootstrap.. Evol.

[29] Nei M, Kumar S, Takahashi K (1998). The optimization principle in phylogenetic analysis tends to give incorrect topologies when the number of nucleotides or amino acids used is small.. Proc Natl Acad Sci U S A.

[30] Zhang D, Hewitt GM (1996). Nuclear integrations: challenges for mitochondrial DNA markers.. Trends Ecol Evol.

[31] Stock DW, Powers DA (1995). The cDNA sequence of the lactate dehydrogenase-a of the spiny dogfish (*Squalus acanthias*): corrections to the amino acid sequence and an analysis of the phylogeny of vertebrate lactate dehydrogenases.. Mol Mar Biol Biotechnol.

[32] Hong J, Salo WL, Chen Y, Atkinson BG, Anderson PM (1996). The promoter region of the carbamoyl-phosphate synthetase iii gene of *Squalus acanthias*.. J Mol Evol.

[33] Salaneck E, Ardell DH, Larson ET, Larhammar D (2003). Three neuropeptide y receptor genes in the spiny dogfish, *Squalus acanthias*, support en bloc duplications in early vertebrate evolution.. Mol Biol Evol.

[34] Kabasakal H (2009). Observations on a rare shark, *Oxynotus centrina* (Chondrichthyes: Oxynotidae), in the sea of marmara (north-western turkey).. Pan-American Journal of Aquatic Sciences.

[35] Stelbrink B, von Rintelen T, Cliff G, Kriwet J (2010). Molecular systematics and global phylogeography of angel sharks (genus Squatina).. Mol Phylogenet Evol.

