# Supplementary material for: Classification of Sharks in the Egyptian Mediterranean Waters Using Morphological and DNA Barcoding Approaches
Source: PLoS One. 2011 Nov 2;6(11):e27001. doi: 10.1371/journal.pone.0027001 (PMC3206905; doi:10.1371/journal.pone.0027001)
Supplement: Figure S1 — Specimens' pictures for each species under study. A: Squalus acanthias; B: Oxynotus centrina; C: Squatina squatina; D: Scyliorhinus canicula; E: Scyliorhinus stellaris; F: Mustelus mustelus; G: Mustelus punctulatus and H: Carcharhinus altimus. (PDF) [file pone.0027001.s001.pdf]

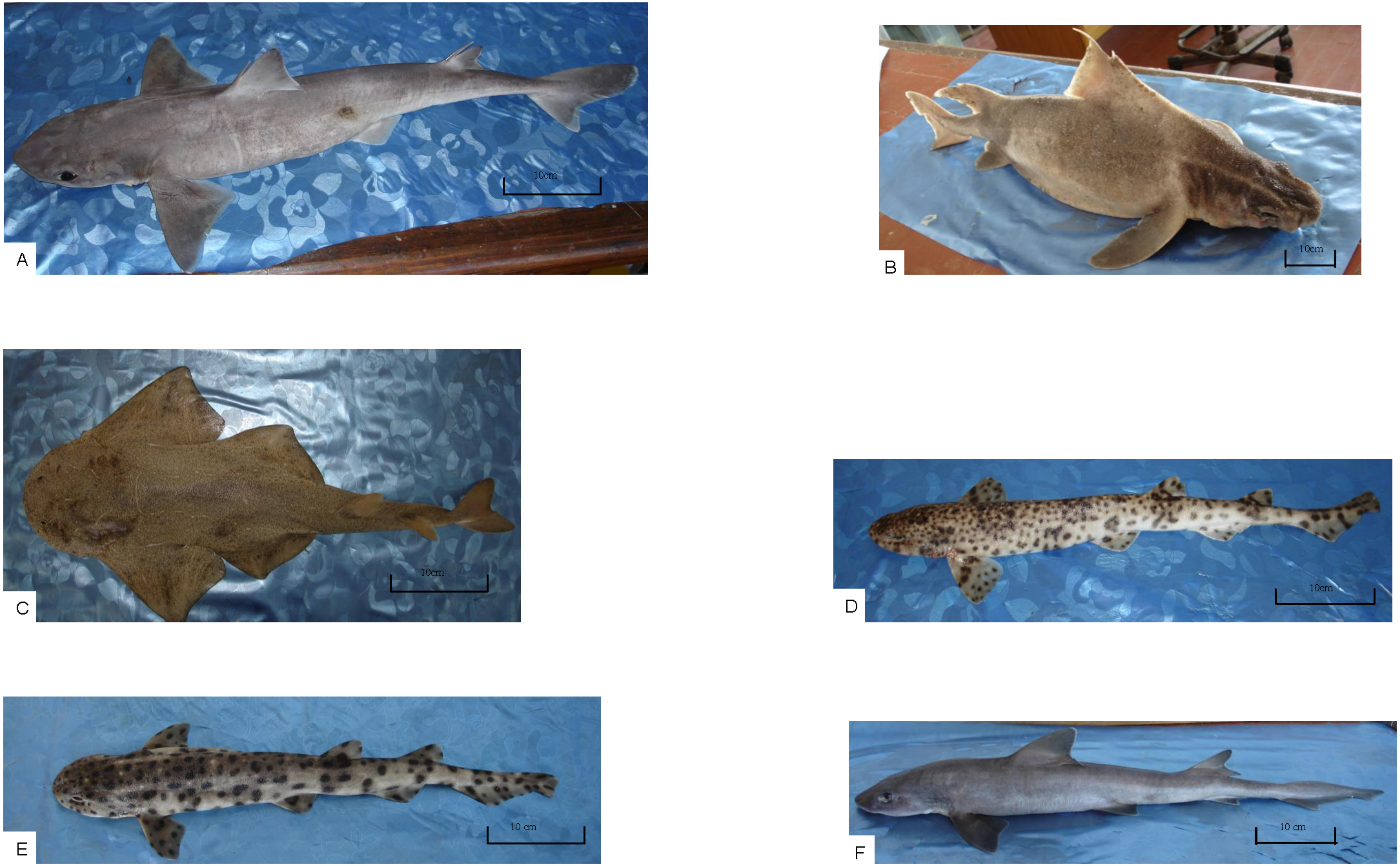
A
10cm
B
10cm
C
10cm
D
10cm
E
10 cm
F
10 cm

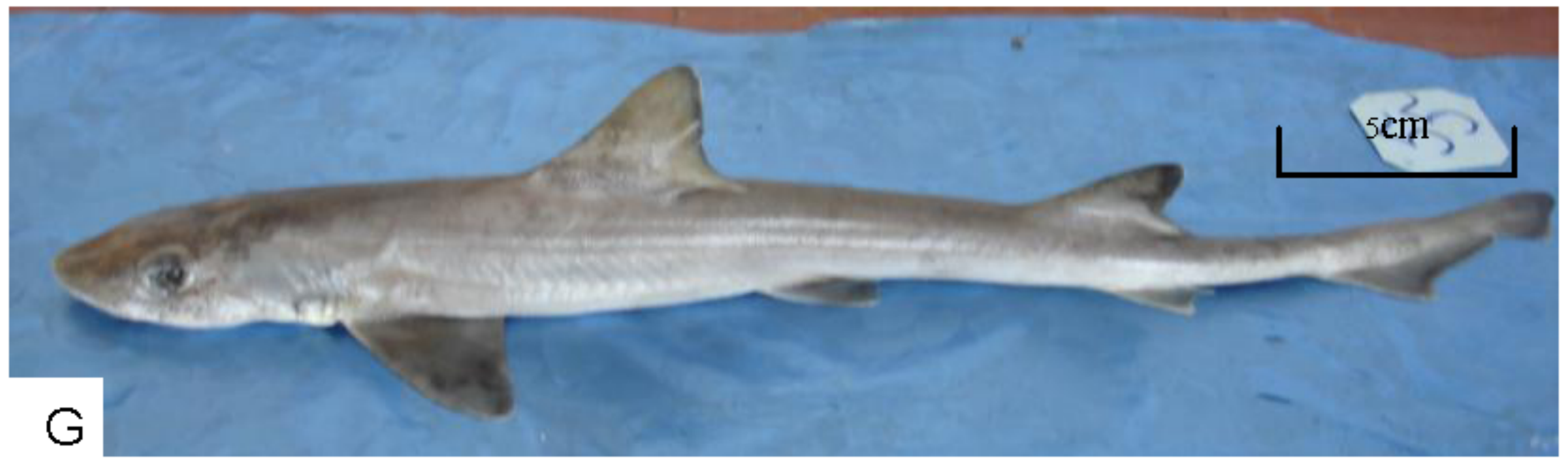

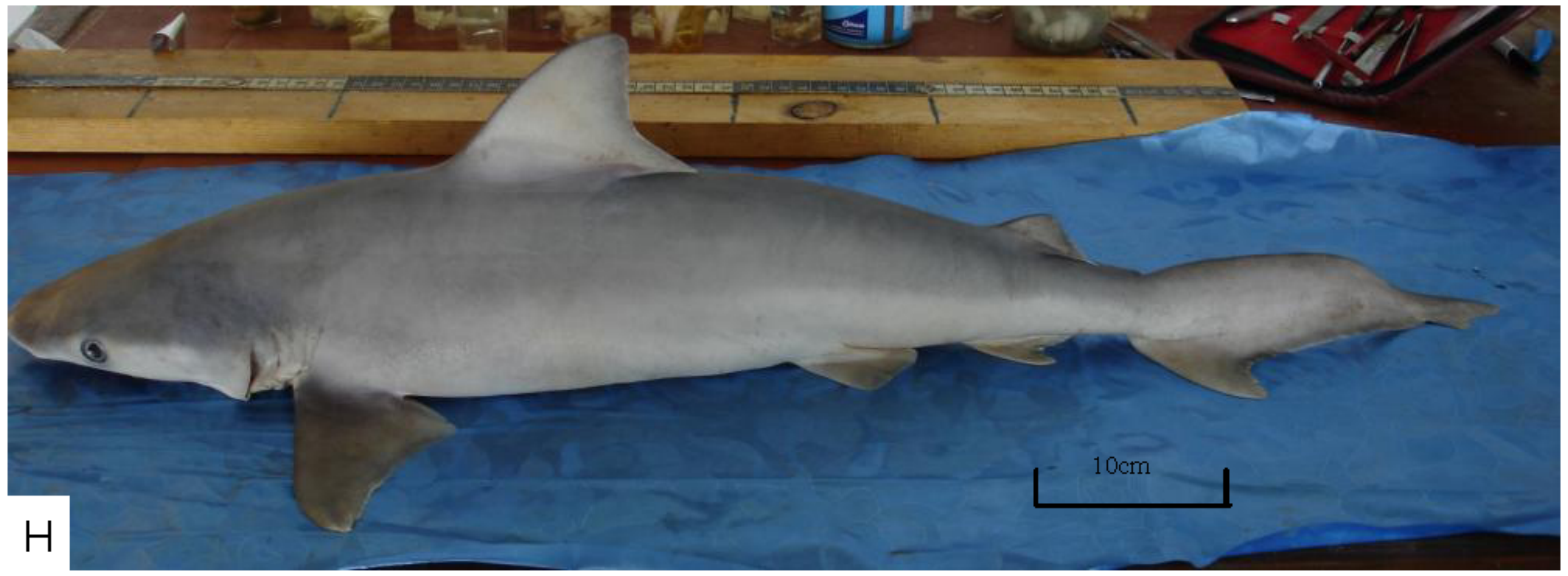

Figure S1: Specimens' pictures for each species under study
A: Squalus acanthias; B: Oxynotus centrina; C: Squatina squatina; D: Scyliorhinus canicula; E: Scyliorhinus stellaris; F: Mustelus mustelus; G: Mustelus punctulatus and H: Carcharhinus altimus.
