# Supplementary material for: Classification of Sharks in the Egyptian Mediterranean Waters Using Morphological and DNA Barcoding Approaches
Source: PLoS One. 2011 Nov 2;6(11):e27001. doi: 10.1371/journal.pone.0027001 (PMC3206905; doi:10.1371/journal.pone.0027001)
Supplement: Table S1 — Sequence analysis results: maximum identity using GenBank database, first and second best similarities using BOLD identification engine. X: indicates specimen checked for best match using BOLD identification engine, and the site could not match and diverted it to GenBank database. Consensus identification was decided depending on best similarity for the specimen, using both GenBank database and BOLD identification engine, putting into consideration the morphological and morphometric measurements. (PDF) [file pone.0027001.s002.pdf]

Sample no.	Test no.	GenBank		BOLD				Consensus identification
		Matched species	Max identity %	First best matched species	Similarity %	2nd best matched species	Similarity %	
1	Test (1)	*Mustelus mustelus*	97	*Mustelus mustelus*	98.31	*Mustelus mustelus*	98.26	*Mustelus mustelus*
	Test (2)	*Mustelus mustelus*	100	*Mustelus mustelus*	100	*Mustelus mustelus*	100	
	Test (3)	*Mustelus mustelus*	99	*Mustelus mustelus*	99.83	*Mustelus mustelus*	99.82	
2	Test (1)	*Mustelus mustelus*	100	*Mustelus mustelus*	100	*Mustelus mustelus*	100	*Mustelus mustelus*
	Test (2)	*Mustelus mustelus*	100	*Mustelus mustelus*	100	*Mustelus mustelus*	99.65	
	Test (3)	*Mustelus mustelus*	100	*Mustelus mustelus*	100	*Mustelus mustelus*	99.69	
3	Test (1)	*Mustelus mustelus*	100	*Mustelus mustelus*	100	*Mustelus mustelus*	100	*Mustelus mustelus*
	Test (2)	*Mustelus mustelus*	100	*Mustelus mustelus*	100	*Mustelus mustelus*	100	
	Test (3)	*Mustelus mustelus*	100	*Mustelus mustelus*	100	*Mustelus mustelus*	100	
5	Test (1)	*Mustelus mustelus*	100	*Mustelus mustelus*	100	*Mustelus mustelus*	100	*Mustelus mustelus*
	Test (2)	*Mustelus mustelus*	100	*Mustelus mustelus*	100	*Mustelus mustelus*	100	
	Test (3)	*Mustelus mustelus*	100	*Mustelus mustelus*	100	*Mustelus mustelus*	100	
6	Test (1)	*Mustelus henlei*	85	*Mustelus punctulatus*	100	*Mustelus punctulatus*	100	*Mustelus punctulatus*
	Test (2)	*Mustelus sp.*	100	*Mustelus punctulatus*	100	*Mustelus punctulatus*	100	
	Test (3)	*Mustelus sp.*	95	X	X	X	X	
7	Test (1)	*Mustelus sp.*	99	*Mustelus punctulatus*	99.51	*Mustelus punctulatus*	99.51	*Mustelus punctulatus*
	Test (2)	*Mustelus sp.*	100	*Mustelus punctulatus*	100	*Mustelus punctulatus*	100	
	Test (3)	*Mustelus sp.*	99	*Mustelus punctulatus*	99.84	*Mustelus punctulatus*	99.84	
8	Test (1)	*Mustelus sp.*	100	*Mustelus punctulatus*	100	*Mustelus punctulatus*	100	*Mustelus punctulatus*
	Test (2)	*Mustelus sp.*	99	*Mustelus punctulatus*	99.84	*Mustelus punctulatus*	99.84	
	Test (3)	*Mustelus sp.*	99	*Mustelus sp*. zpl 00058	99.82	*Mustelus punctulatus*	99.69	
9	Test (1)	*Mustelus sp.*	87	*Mustelus punctulatus*	99.05	*Mustelus punctulatus*	99.05	*Mustelus punctulatus*
	Test (2)	Zebrafish	100	X	X	X	X	
	Test (3)	*Mustelus sp.*	100	*Mustelus sp*. zpl 00058	100	*Mustelus punctulatus*	99.68	
10	Test (1)	*Mustelus mustelus*	100	*Mustelus mustelus*	100	*Mustelus mustelus*	100	*Mustelus mustelus*
	Test (2)	*Mustelus mustelus*	100	*Mustelus mustelus*	100	*Mustelus mustelus*	99.84	
	Test (3)	*Mustelus mustelus*	99	*Mustelus mustelus*	98.75	*Mustelus mustelus*	98.65	
11	Test (1)	*Mustelus sp.*	99	*Mustelus* sp. zpl 00058	99.29	*Mustelus punctulatus*	98.9	*Mustelus punctulatus*
	Test (2)	*Mustelus sp*	100	*Mustelus punctulatus*	100	*Mustelus punctulatus*	100	
	Test (3)	*Mustelus sp.*	100	*Mustelus sp*. zpl 00058	100	*Mustelus punctulatus*	99.84	

Sample no.	Test no.	GenBank		BOLD				Consensus identification
		Matched species	Max identity %	First best matched species	Similarity %	2nd best matched species	Similarity %	
12	Test (1)	*Mustelus sp.*	100	*Mustelus punctulatus*	100	*Mustelus punctulatus*	100	*Mustelus punctulatus*
	Test (2)	*Mustelus sp*	100	*Mustelus punctulatus*	100	*Mustelus punctulatus*	100	
	Test (3)	*Mustelus sp.*	96	X	X	X	X	
13	Test (1)	*Mustelus lunulatus*	92	X	X	X	X	*Mustelus mustelus*
	Test (2)	*Mustelus mustelus*	100	*Mustelus mustelus*	100	*Mustelus mustelus*	100	
	Test (3)	*Mustelus mustelus*	100	*Mustelus mustelus*	100	*Mustelus mustelus*	99.84	
14	Test (1)	*Mustelus mustelus*	87	*Mustelus mustelus*	97.36	*Mustelus mustelus*	97.36	*Mustelus mustelus*
	Test (2)	*Mustelus mustelus*	100	*Mustelus mustelus*	100	*Mustelus mustelus*	99.84	
	Test (3)	*Mustelus mustelus*	79	X	X	*X*	X	
16	Test (1)	*Mustelus mustelus*	100	*Mustelus mustelus*	100	*Mustelus mustelus*	100	*Mustelus mustelus*
	Test (2)	*Mustelus mustelus*	99	*Mustelus mustelus*	99.82	*Mustelus mustelus*	99.69	
	Test (3)	*Mustelus mustelus*	99	*Mustelus mustelus*	99.82	*Mustelus mustelus*	99.48	
17	Test (2)	*Squalus sp.*	99	*Squalus acanthias*	100	*Squalus acanthias*	100	*Squalus acanthias*
	Test (3)	*Squalus sp.*	99	*Squalus acanthias*	99.84	*Squalus blainville*	99.84	
18	Test (2)	*Squalus sp.*	99	*Squalus acanthias*	99.83	*Squalus blainville*	99.53	*Squalus acanthias*
	Test (3)	*Squalus sp.*	99	*Squalus acanthias*	100	*Squalus blainville*	99.68	
19	Test (2)	*Squalus megalops*	82	*Squalus acanthias*	98.7(solid)	*Squalus blainville*	98.31	*Squalus acanthias*
	Test (3)	*Squalus megalops*	99	*Squalus blainville*	99.84	*Squalus blainville*	99.84	
20	Test (2)	*Squalus sp.*	81	X	X	X	X	*Squalus acanthias*
	Test (3)	*Squalus megalops*	99	*Squalus acanthias*	100	*Squalus acanthias*	100	
22	Test (2)	*Squalus sp.*	99	*Squalus acanthias*	99.84	*Squalus blainville*	99.84	*Squalus acanhias*
	Test (3)	*Hippocampus angustus*	76	X	X	X	X	
25	Test (1)	*Scyliorhinus stellaris*	100	*Scyliorhinus stellaris*	100(solid)	*Scyliorhinus stellaris*	99.51	*Scyliorhinus stellaris*
	Test (2)	*Gallus gallus*	87	X	X	X	X	
	Test (3)	*Scyliorhinus stellaris*	99	*Scyliorhinus stellaris*	100(solid)	*Scyliorhinus stellaris*	99.47	
26	Test (1)	*Scyliorhinus stellaris*	86	X	X	X	X	*Scyliorhinus stellaris*
	Test (2)	*Scyliorhinus stellaris*	99	*Scyliorhinus stellaris*	100(solid)	*Scyliorhinus stellaris*	99.5	
	Test (3)	*Scyliorhinus stellaris*	100	*Scyliorhinus stellaris*	100(solid)	*Scyliorhinus stellaris*	99.51	
27	Test (1)	*Scyliorhinus stellaris*	100	*Scyliorhinus stellaris*	100(solid)	*Scyliorhinus stellaris*	99.51	*Scyliorhinus stellaris*
	Test (2)	*Scyliorhinus stellaris*	100	*Scyliorhinus stellaris*	100(solid)	*Scyliorhinus stellaris*	99.51	
	Test (3)	*Scyliorhinus stellaris*	99	*Scyliorhinus stellaris*	100(solid)	*Scyliorhinus stellaris*	99.5	
28	Test (1)	*Scyliorhinus stellaris*	100	*Scyliorhinus stellaris*	100(solid)	*Scyliorhinus stellaris*	99.51	*Scyliorhinus stellaris*
	Test (2)	*Scyliorhinus stellaris*	100	*Scyliorhinus stellaris*	100(solid)	*Scyliorhinus stellaris*	99.51	
	Test (3)	*Mus musculus*	96	X	X	X	X	

Sample no.	Test no.	GenBank		BOLD				Consensus identification
		Matched species	Max identity %	First best matched species	Similarity%	2nd best matched species	Similarity %	
29	Test (1)	*Scyliorhinus canicula*	99	*Scyliorhinus stellaris*	99.33(solid)	*Scyliorhinus stellaris*	98.86	*Scyliorhinus canicula*
	Test (2)	*Scyliorhinus canicula*	99	*Scyliorhinus stellaris*	99.84	*Scyliorhinus stellaris*	99.84	
	Test (3)	*Scyliorhinus canicula*	99	*Scyliorhinus stellaris*	100	*Scyliorhinus stellaris*	100	
30	Test (1)	*Scyliorhinus canicula*	99	*Scyliorhinus canicula*	100	*Scyliorhinus stellaris*	99.84	*Scyliorhinus canicula*
	Test (2)	*Scyliorhinus canicula*	99	*Scyliorhinus canicula*	100	*Scyliorhinus stellaris*	99.84	
	Test (3)	*Scyliorhinus canicula*	98	*Scyliorhinus canicula*	98.83	*Scyliorhinus stellaris*	99.67	
31	Test (1)	*Scyliorhinus canicula*	99	*Scyliorhinus stellaris*	100	*Scyliorhinus stellaris*	100	*Scyliorhinus canicula*
	Test (2)	*Scyliorhinus stellaris*	74	X	X	X	X	
	Test (3)	marine zooplankton	79	X	X	X	X	
32	Test (1)	*Scyliorhinus canicula*	99	*Scyliorhinus stellaris*	100	*Scyliorhinus stellaris*	100	*Scyliorhinus canicula*
	Test (2)	*Scyliorhinus canicula*	99	*Scyliorhinus stellaris*	100	*Scyliorhinus stellaris*	100	
	Test (3)	*Scyliorhinus canicula*	99	*Scyliorhinus stellaris*	100	*Scyliorhinus stellaris*	100	
33	Test (1)	*Scyliorhinus canicula*	99	*Scyliorhinus canicula*	100	*Scyliorhinus canicula*	100	*Scyliorhinus canicula*
	Test (2)	*Scyliorhinus canicula*	99	*Scyliorhinus canicula*	100	*Scyliorhinus canicula*	100	
	Test (3)	*Scyliorhinus canicula*	99	*Scyliorhinus canicula*	100	*Scyliorhinus canicula*	100	
34	Test (1)	*Scyliorhinus canicula*	79	X	X	X	X	*Scyliorhinus canicula*
	Test (2)	*Scyliorhinus canicula*	99	*Scyliorhinus stellaris*	100	*Scyliorhinus stellaris*	100	
	Test (3)	*Bos taurus* REX2	100	X	X	X	X	
35	Test (1)	*Scyliorhinus canicula*	99	*Scyliorhinus canicula*	99.84	*Scyliorhinus canicula*	99.84	*Scyliorhinus canicula*
	Test (2)	*Scatophagus argus*	76	X	X	X	X	
	Test (3)	*Scyliorhinus canicula*	99	*Scyliorhinus canicula*	99.84	*Scyliorhinus canicula*	99.84	
36	Test (1)	*Scyliorhinus canicula*	99	*Scyliorhinus stellaris*	98.69(solid)	*Scyliorhinus stellaris*	98.69	*Scyliorhinus canicula*
	Test (2)	*Scatophagus argus*	82	X	X	X	X	
	Test (3)	*Scyliorhinus canicula*	99	*Scyliorhinus stellaris*	100	*Scyliorhinus stellaris*	100	
37	Test (1)	*Scyliorhinus canicula*	99	*Scyliorhinus canicula*	100	*Scyliorhinus canicula*	100	*Scyliorhinus canicula*
	Test (2)	*Scyliorhinus canicula*	99	*Scyliorhinus canicula*	100	*Scyliorhinus canicula*	100	
	Test (3)	*Scyliorhinus canicula*	99	*Scyliorhinus canicula*	100	*Scyliorhinus canicula*	99.69	
38	Test (1)	*Scyliorhinus canicula*	98	*Scyliorhinus stellaris*	99.18	*Scyliorhinus stellaris*	99.18	*Scyliorhinus canicula*
	Test (2)	*Scyliorhinus canicula*	99	*Scyliorhinus stellaris*	99.84	*Scyliorhinus stellaris*	99.84	
	Test (3)	*Scyliorhinus canicula*	99	*Scyliorhinus stellaris*	99.84	*Scyliorhinus stellaris*	99.84	
39	Test (1)	*Scyliorhinus canicula*	98	*Scyliorhinus canicula*	99.38	*Scyliorhinus canicula*	99.38	*Scyliorhinus canicula*
	Test (2)	*Scyliorhinus canicula*	99	*Scyliorhinus canicula*	100	*Scyliorhinus canicula*	100	
	Test (3)	*Scyliorhinus canicula*	97	*Scyliorhinus canicula*	97.9	*Scyliorhinus canicula*	97.9	

Sample no.	Test no.	GenBank		BOLD				Consensus identification
		Matched species	Max identity %	First best matched species	Similarity%	2nd best matched species	Similarity %	
40	Test (1)	*Scyliorhinus canicula*	99	*Scyliorhinus canicula*	100	*Scyliorhinus canicula*	100	*Scyliorhinus canicula*
	Test (2)	*Scyliorhinus canicula*	99	*Scyliorhinus canicula*	100	*Scyliorhinus canicula*	100	
	Test (3)	*Halichoeres prosopeion*	86	X	X	X	X	
41	Test (1)	*Scyliorhinus canicula*	99	*Scyliorhinus canicula*	99.84	*Scyliorhinus canicula*	99.84	*Scyliorhinus canicula*
	Test (2)	*Scyliorhinus canicula*	97	*Scyliorhinus canicula*	98.55	*Scyliorhinus canicula*	98.55	
	Test (3)	*Allocapnia granulata*	73	X	X	X	X	
42	Test (1)	*Scyliorhinus canicula*	99	*Scyliorhinus canicula*	100	*Scyliorhinus canicula*	100	*Scyliorhinus canicula*
	Test (2)	*Scyliorhinus canicula*	99	*Scyliorhinus canicula*	100	*Scyliorhinus canicula*	100	
	Test (3)	*Scyliorhinus canicula*	99	*Scyliorhinus canicula*	100	*Scyliorhinus canicula*	100	
43	Test (1)	*Scyliorhinus canicula*	99	*Scyliorhinus canicula*	99.84	*Scyliorhinus canicula*	99.84	*Scyliorhinus canicula*
	Test (2)	*Scyliorhinus canicula*	99	*Scyliorhinus canicula*	99.84	*Scyliorhinus canicula*	99.84	
	Test (3)	*Scyliorhinus canicula*	99	*Scyliorhinus canicula*	99.84	*Scyliorhinus canicula*	99.84	
44	Test (1)	*Scyliorhinus canicula*	99	*Scyliorhinus canicula*	100	*Scyliorhinus canicula*	100	*Scyliorhinus canicula*
	Test (2)	*Scyliorhinus canicula*	99	*Scyliorhinus canicula*	100	*Scyliorhinus canicula*	100	
	Test (3)	*Scyliorhinus canicula*	96	*Scyliorhinus stellaris*	97	*Scyliorhinus stellaris*	97	
45	Test (1)	*Scyliorhinus canicula*	99	*Scyliorhinus canicula*	99.84	*Scyliorhinus canicula*	99.84	*Scyliorhinus canicula*
	Test (2)	*Scyliorhinus canicula*	81	X	X	X	X	
	Test (3)	*Scyliorhinus canicula*	90	X	X	X	X	
46	Test (1)	*Scyliorhinus canicula*	99	*Scyliorhinus canicula*	100	*Scyliorhinus canicula*	100	*Scyliorhinus canicula*
	Test (3)	*Scyliorhinus canicula*	91	X	X	X	X	
47	Test (1)	*Scyliorhinus canicula*	99	*Scyliorhinus canicula*	100	*Scyliorhinus canicula*	100	*Scyliorhinus canicula*
	Test (2)	*Scyliorhinus canicula*	99	*Scyliorhinus canicula*	100	*Scyliorhinus canicula*	100	
	Test (3)	*Scyliorhinus canicula*	99	*Scyliorhinus canicula*	100	*Scyliorhinus canicula*	100	
48	Test (1)	*Scyliorhinus canicula*	99	*Scyliorhinus canicula*	100	*Scyliorhinus canicula*	100	*Scyliorhinus canicula*
	Test (2)	*Scyliorhinus canicula*	99	*Scyliorhinus canicula*	100	*Scyliorhinus canicula*	100	
	Test (3)	*Scyliorhinus canicula*	99	*Scyliorhinus canicula*	100	*Scyliorhinus canicula*	100	
49	Test (1)	*Scyliorhinus canicula*	99	*Scyliorhinus canicula*	100	*Scyliorhinus canicula*	100	*Scyliorhinus canicula*
	Test (2)	*Scyliorhinus canicula*	99	*Scyliorhinus canicula*	99.84	*Scyliorhinus canicula*	99.84	
	Test (3)	*Scyliorhinus canicula*	99	*Scyliorhinus canicula*	100	*Scyliorhinus canicula*	100	
50	Test (1)	*Scyliorhinus canicula*	99	*Scyliorhinus canicula*	99.84	*Scyliorhinus canicula*	99.84	*Scyliorhinus canicula*
	Test (2)	*Scyliorhinus canicula*	99	*Scyliorhinus canicula*	99.84	*Scyliorhinus canicula*	99.84	
	Test (3)	*Scyliorhinus canicula*	99	*Scyliorhinus canicula*	99.84	*Scyliorhinus canicula*	99.84	
51	Test (1)	*Squatina squatina*	85	*Squatina* squatina	99.17(solid)	*Squatina* squatina	99.17	*Squatina squatina*
	Test (2)	*Squatina squatina*	99	*Squatina* squatina	99.52(solid)	*Squatina* squatina	99.36	
	Test (3)	*Squatina squatina*	98	*Squatina* squatina	98.53(solid)	*Squatina* squatina	98.37	

Sample no.	Test no.	GenBank		BOLD				Consensus identification
		Matched species	Max identity %	First best matched species	Similarity%	2nd best matched species	Similarity %	
52	Test (1)	*Squatina squatina*	99	*Squatina* squatina	99.53(solid)	*Squatina* squatina	99.37	*Squatina squatina*
	Test (2)	*Squatina squatina*	98	*Squatina* squatina	99.51(solid)	*Squatina* squatina	99.34	
	Test (3)	*Squatina squatina*	98	*Squatina* squatina	99.18(solid)	*Squatina* squatina	99.01	
53	Test (1)	*Squatina squatina*	84	*Squatina* squatina	99.42(solid)	*Squatina* squatina	99.42	*Squatina squatina*
	Test (2)	*Squatina squatina*	98	*Squatina* squatina	99.84(solid)	*Squatina squatina*	99.67	
	Test (3)	*Squatina squatina*	99	*Squatina* squatina	99.68(solid)	*Squatina* squatina	99.52	
54	Test (1)	*Carcharhinus altimus*	99	*Carcharhinus plumbeus*	99.53	*Carcharhinus plumbeus*	99.53	*Carcharhnius atitmus*
	Test (2)	*Carcharhinus altimus*	99	*Carcharhinus plumbeus*	99.53	*Carcharhinus plumbeus*	99.53	
	Test (3)	*Carcharhinus altimus*	99	*Carcharhinus plumbeus*	99.84	*Carcharhinus plumbeus*	99.84	
55	Test (1)	*Carcharhinus altimus*	99	*Carcharhinus plumbeus*	99.84	*Carcharhinus plumbeus*	99.84	*Carcharhinus altimus*
	Test (2)	*Carcharhinus altimus*	85	X	X	X	X	
56	Test (2)	*Centroscymnus coelolepis*	92	*Oxynotus* centrina	100	*Oxynotus centrina*	100	*Oxynotus centrina*
	Test (3)	*Centroscymnus coelolepis*	92	*Oxynotus* centrina	100(solid)	*Oxynotus* centrina	100	

Table S1: Sequence analysis results: maximum identity using GenBank database, first and second best similarities using BOLD identification engine.

X : indicates specimen checked for best match using BOLD identification engine, and the site could not match and diverted it to GenBank database.
Consensus identification was decided depending on best similarity for the specimen, using both GenBank database and BOLD identification engine, putting into consideration the morphological and morphometric measurements.
